# Mindfulness Development via Biofeedback for Anxiety in Hospitalized Patients: A Randomized Controlled Trial

**DOI:** 10.3390/brainsci16070748

**Published:** 2026-07-15

**Authors:** Anastasia V. Kotelnikova, Vera M. Ruzinova, Maria G. Kiseleva, Beatrice Volel, Anastasia A. Kukshina

**Affiliations:** 1 Sechenov University, Moscow 119048, Russia; 2 Research Institute for Healthcare Organization and Medical Management, Moscow Department of Healthcare, Moscow 115088, Russia

**Keywords:** mindfulness, anxiety disorders, biofeedback, BFB

## Abstract

**Highlights:**

**What are the main findings?**

- Biofeedback (BFB) training, either alone or combined with pharmacotherapy, produced significantly greater reductions in anxiety symptoms (HAM-A) compared to medication monotherapy in patients with anxiety disorders.
- BFB-based mindfulness enhancing was associated with notable increases in overall mindfulness (FFMQ) and its key components (observing, describing, acting with awareness, non-reactivity), which were maintained at one month, whereas medication monotherapy was associated with decreased mindfulness scores.

**What are the implications of the main findings?**

- BFB may serve as an effective non-pharmacological intervention for anxiety disorders, targeting both symptom reduction and the development of psychological resources (mindfulness).
- The integration of BFB into standard treatment protocols could offer a comprehensive approach that addresses both the neurophysiological correlates and cognitive-affective aspects of anxiety, potentially contributing to more sustainable therapeutic outcomes.

**Abstract:**

**Background**: Anxiety disorders are prevalent and debilitating. Given challenges in maintaining mindfulness at one-month remission, integrative approaches targeting anxiety and psychological resources (especially mindfulness) have gained interest. This study aimed to assess the effectiveness of adding biofeedback to the treatment of anxiety disorders. **Methods**: Inpatients with anxiety disorder (ICD10 F40/F41) were randomized to three groups: (1) biofeedback for enhancing mindfulness (BFB, n = 76); (2) medication therapy (MT, escitalopram 10 mg/day, n = 46); and (3) combined therapy (BFB + MT, CT, n = 66). Per-protocol analysis: 180 completers; ITT: all 188 randomized patients. The primary outcomes were changes in the Hamilton Anxiety Rating Scale (HAM-A) total score and the Five Facet Mindfulness Questionnaire total score, assessed at baseline, after 10 days of treatment, and at one-month follow-up. The HAM-A assessor was blinded to group allocation. **Results**: In the per-protocol analysis, the BFB and CT groups showed a significantly greater reduction in the HAM-A score than the MT group did. For example, post-treatment, the mean difference between BFB and MT groups was –6.30 points (95% CI: –9.12 to –3.48, *p* = 0.00046). The BFB and CT groups did not differ significantly. For mindfulness, the BFB and CT groups showed an increase, whereas the MT group showed a decrease (BFB vs. MT: mean difference 30.28 points, 95% CI: 23.54–37.02, *p* < 0.000001). Intention-to-treat (ITT) analysis (n = 188) confirmed the robustness of these findings. **Conclusions**: BFB appears to be a promising approach for reducing anxiety and fostering mindfulness skills; these findings suggest it may be worth exploring further as a potential component of comprehensive therapy for anxiety disorders. **Clinical Trial Registration**: ClinicalTrials.gov NCT07628153 (retrospectively registered). The trial was retrospectively registered, and the primary analysis was conducted on a per-protocol population.

## 1. Introduction

Anxiety disorders (ADs) remain one of the most prevalent forms of mental pathology, exerting a significant negative impact on patients’ quality of life and creating a substantial burden on healthcare systems [1,2]. Anxiety is most commonly and effectively treated with psychopharmacotherapy [3]. Despite the existence of proven pharmacotherapeutic approaches, the challenge of achieving and maintaining one-month remission and preventing relapse remains relevant [4]. In this context, there is growing interest in integrative approaches aimed at alleviating symptoms and developing patients’ internal psychological resources, primarily mindfulness skills [5].

Mindfulness, understood as the ability to pay purposeful, non-judgmental attention to the present moment, is recognized as a key factor in psychological flexibility and emotional regulation [6,7]. Its development correlates with a reduction in anxiety severity through decentering, acceptance, and reduced reactivity [8,9]. The Five Facet Mindfulness Model operationalizes this construct through components such as observing, describing, acting with awareness, non-judgment of inner experience, and non-reactivity [10], allowing for a differentiated assessment of the effects of psychotherapeutic interventions.

Biofeedback (BFB) is a promising tool for the targeted development of mindfulness skills. Its psychological impact is based on overcoming “physiological blindness”: providing the patient with real-time objective information about hidden physiological processes (heart rate, EEG rhythms, etc.) creates a “neurophysiological mirror” effect [11]. This contributes to the formation of conscious control, increased self-efficacy, and overcoming learned helplessness—key targets in anxiety therapy [12,13]. For the treatment of anxiety, a number of studies have demonstrated the high clinical effectiveness of biofeedback [14,15].

However, to our knowledge, no study has directly compared biofeedback with pharmacotherapy in a head-to-head design, nor examined its effects on specific facets of mindfulness as measured by the FFMQ. Our study therefore addresses this gap.

The aim was to compare, in a superiority design, the effectiveness of biofeedback, combined therapy (biofeedback plus medication), and MT alone on changes in anxiety severity (Hamilton Anxiety Rating Scale (HAM-A) total score) and mindfulness skills (Five Facet Mindfulness Questionnaire (FFMQ) total score) from baseline to immediately after treatment and at one-month follow-up, and to assess the associated benefits and potential harm.

## 2. Materials and Methods

The study procedure complied with ethical standards and was approved by the Local Ethics Committee of I.M. Sechenov First Moscow State Medical University (Protocol No. 14-12 dated 7 July 2022). All participants were informed of the study aims and objectives and voluntary written informed consent was obtained. The study was conducted between July 2022 and February 2025.

Of the 188 randomized patients, 180 completed the study and were included in the per-protocol analysis. These were inpatients with a newly diagnosed anxiety disorder (ICD 10 codes F40 and F41) undergoing treatment at the Department of Psychotherapy of the A.Ya. Kozhevnikov Clinic of Nervous Diseases at the Sechenov University Clinical Center. Specific subtypes (e.g., panic disorder and GAD) were not recorded and no subgroup analyses by diagnosis were performed. Diagnoses were established by a board-certified psychiatrist according to ICD 10 criteria using a clinical interview (no structured diagnostic interview was employed). Comorbid psychotic disorders, substance use disorders, or organic brain syndromes were exclusionary. The sample consisted of 68.9% women and 31.1% men, aged 19–74 years (M = 43.2; Me = 42.0; SD = 13.6). All patients had mild–moderate functional impairment according to the International Classification of Functioning, Disability, and Health (ICF), corresponding to a mild or moderate class of limitations. The exclusion criteria were as follows: (1) photosensitive epilepsy, (2) severe intellectual, attentional, or memory impairment, (3) exacerbation of mental illness, (4) lack of motivation, and (5) unwillingness or inability to provide written consent. The reasons for exclusion during treatment were as follows: (1) exacerbation of a severe mental and/or somatic disease; (2) withdrawal of consent; (3) inability to attend one or more individual sessions; (4) refusal to do homework; and (5) inability to complete all three assessments. Motivation was assessed clinically during the initial interview; patients who expressed hesitation or unwillingness to adhere to the protocol were not enrolled. The voluntary nature of participation and the right to withdraw at any time were emphasized in the informed consent document.

This was an open-label, randomized, parallel-group study based on simple (unrestricted) randomization with an equal probability of assignment to each group without blocks or stratification. The allocation sequence was generated by an independent programmer using a Microsoft Excel random number generator and was concealed in opaque, sequentially numbered, sealed envelopes. The envelopes were opened by the enrolling clinicians (a psychiatrist and a medical psychologist) only after the patient had been deemed eligible and had provided written informed consent, thereby ensuring allocation concealment throughout recruitment. Initially, 188 patients were included in this study. During treatment, eight patients from the combined therapy (CT) group were excluded from the analysis according to predefined criteria: missing more than two individual sessions (n = 5), refusal to complete homework (n = 2), and non-adherence to the session protocol (n = 1). To evaluate potential bias from the eight excluded participants (all from the CT group), we compared their baseline age, sex, HAM-A total, and FFMQ total with those of the 58 CT completers using the Mann–Whitney U test and Fisher’s exact test; none of the differences were statistically significant (all *p* > 0.10). The final per-protocol completer analysis included 180 patients who completed the study and were distributed as follows: BFB group, 76; CT group, 58; MT group, 46. The unequal group sizes resulted from simple randomization and protocol-driven exclusions, and the protocol remained unchanged throughout the study. We chosen unrestricted randomization to maintain simplicity and avoid potential selection bias. Although this resulted in unequal group sizes, our statistical methods (repeated measures MANOVA/ANOVA) are robust to moderate imbalances, and baseline characteristics were comparable across groups.

To avoid bias in the assessment of anxiety using the HAM-A at the post-treatment (after 10 days) and follow-up (one month) stages, an independent psychiatrist who was unaware of the group allocation and who did not participate in the therapeutic interventions was invited to perform the diagnostics. To reduce variability, all HAM-A assessors were trained to use a standardized interview technique. Baseline anxiety (before treatment) was assessed by a treating psychiatrist, after which the patients were randomized, and all subsequent assessments were performed by an independent specialist. The statistician performing data analysis was not informed of the study hypotheses.

Patients were randomized into three groups: Group 1, biofeedback for enhancing mindfulness (BFB group, n = 76); Group 2, medication therapy (MT group, n = 46); and Group 3, MT combined with BFB (combined therapy, CT group, n = 58). In the BFB group, 73.7% were women and 26.3% were men, aged 21–67 years (M = 40.8, Me = 42.0, SD = 10.1). In the MT group, 60.9% were women and 39.1% were men, aged 21–71 years (M = 46.5; Me = 43.0; SD = 14.2). In the CT group, 69.0% were women and 31.0% were men, aged 19–74 years (M = 43.6; Me = 41.0; SD = 16.2). The groups were statistically comparable for age, H (2, n = 180) = 3.7, *p* = 0.16. For sex, pairwise comparisons using Fisher’s exact test yielded *p* > 0.05.

Medication therapy in the MT and CT groups followed a single protocol according to the clinical recommendations approved by the Ministry of Health of the Russian Federation (level of evidence A, certainty of evidence 1): monotherapy with escitalopram, a selective serotonin reuptake inhibitor, at a standard dose of 10 mg once daily in the morning. No dose titration was performed, and the patient received 10 mg once daily from day 1. Therapy was initiated during the inpatient’s stay and continued at the time of follow-up assessments.

The mindfulness-enhancing program using biofeedback consisted of 10 individual 60 min psychotherapeutic sessions over two working weeks (Monday to Friday, with a weekend break). Training was conducted using the “Reacor” system (TU 9442 006 24176382 2006, Registration Certificate No. FSR 2009/05647, manufactured by Medicom MTD LLC), which records physiological parameters and converts them into auditory and visual signals in real time. After group formation, patients in the BFB and CT groups underwent daily 60 min sessions with a medical psychologist (certified in the BFB method, accredited as a medical psychologist) for 10 working days. Each session consisted of (1) an introduction, homework review, and psychological interventions; (2) a 20 min BFB training session; and (3) assignment of homework. Homework consisted of informational materials for integrating mindfulness practices into daily life, audio recordings of formal meditations, videos of proprioceptive exercises, self-help materials, articles related to the session topic, and a list of daily tasks with spaces for notes. Patients in the MT group were offered the opportunity to complete the BFB program after the study was concluded (for ethical reasons). No concomitant psychotherapy, physiotherapy, or additional psychotropic medications were administered to any patient during the treatment period. Routine medical care and psychiatric consultation (without therapy) were permitted. Adherence was monitored through daily clinical rounds and medical records.

Three assessments were conducted: (1) at baseline, (2) immediately after treatment (after 10 days), and (3) one month after treatment completion. Each assessment involved two stages: psychiatric consultation using psychometric questionnaires and functional diagnostics using the “Medicom MTD” device and the “Reacor” BFB system. The following instruments were used: the Russian version of the HAM-A (1959) [16]—a clinical rating scale comprising the “Somatic anxiety” and “Psychic anxiety” subscales and a total score; and the Russian version of the FFMQ [17], developed by R. Baer [10], which includes the “Observing”, “Describing”, “Acting with awareness”, “Non-judgment of inner experience”, and “Non-reactivity” subscales and a total mindfulness score. Diagnostics using the “Reacor” system include assessment of heart rate and alpha rhythm power (alpha index) in the occipital leads of the brain [18]. The biofeedback protocol was implemented according to an algorithm that included checking the quality of recorded physiological signals, recording the initial background, automatically calculating individual thresholds, performing controlled steps with continuous visual-auditory feedback, recording the final background, and subsequent analysis of the effectiveness of training. Before each controlled stage, the initial background was recorded for a duration of 45–60 s to adjust individual target thresholds. The total duration of the procedure was from 20 to 30 min. The alpha rhythm was recorded using the occipital leads of the EEG (O1, O2) with additional central leads (C3, C4). The controlled parameter was the alpha rhythm power index in the range of 8–13 Hz, calculated in real time after digital signal filtering. The target thresholds were set to 110–120% of the average baseline value (coefficients 1.1–1.2), and the success criterion was considered to be an increase in the average power by at least 15% relative to the baseline level. During the controlled stages, the intensity of auditory feedback varied in proportion to the increase in alpha activity. Fixed percentage thresholds were not used for heart rate: the complex algorithm changed the thresholds individually depending on the dynamics. The controlled stages were accompanied by visual-auditory feedback, the intensity of which was inversely proportional to the current heart rate, which facilitated the development of voluntary cardioregulation skills. The main quantitative indicators were the success index of the managed stage and the success index of the session, reflecting the degree of achievement of the physiological goals of the training and the success of the formation of self-regulation skills. The primary outcome (day 10; one month) was the change in total HAM-A and FFMQ scores from the baseline. Secondary outcomes included the HAM-A and FFMQ subscale scores as well as physiological parameters. HAM-A was assessed by an independent psychiatrist blinded to group allocation (as described above). Patients completed the FFMQ. Physiological parameters were recorded by a technician who was not involved in treatment. The group–time interaction for these measures was also considered primary to assess differential trajectories.

Adverse events were assessed by spontaneous patient reporting during daily clinical rounds. No systematic questionnaire was administered. Events were coded by severity (mild/moderate/severe) and relationship to intervention (unlikely/possible/probable).

The patients and public were not involved in the development of the research question, study design, conduct, or dissemination of the results.

The data supporting the findings of this study are available from the corresponding author upon reasonable request, subject to institutional policies. No publicly archived datasets were generated or analyzed during this study.

Statistical analyses were performed using Statistica 10.0. The following analyses were used: descriptive statistics (mean and standard deviation), significance testing for differences in quantitative variables in independent groups, Z-standardization of data, repeated-measures multivariate analysis of variance (MANOVA), and repeated-measures univariate analysis of variance (ANOVA).

Because the primary outcomes (total HAM-A and FFMQ scores) were prespecified and all other measures were considered secondary/exploratory, no global correction for multiple comparisons across different outcomes was applied, increasing the risk of errors of the first type; thus, the results should be interpreted as preliminary and requiring independent repetition. For univariate repeated-measures ANOVA, the sphericity assumption was tested using Mauchly’s test. When sphericity was violated (*p* < 0.05), the Greenhouse–Geisser correction was applied to adjust the degrees of freedom. For post hoc pairwise comparisons, the Scheffé test, which controls the Type I error rate within each ANOVA, was used. All post hoc analyses were exploratory and not prespecified. The results of the secondary outcomes should be interpreted with caution and require confirmation in future studies.

The analysis was conducted using a complete sample (patients who completed the study). Only completers were included in the analysis of the primary protocol. For the sensitivity ITT analysis, missing post-treatment and follow-up values for the eight excluded CT participants were imputed using a worst-case scenario (baseline value carried forward). No other missing data were observed. The significance level was set at *p* < 0.05. For very small *p*-values (less than 0.000001), the exact *p*-value has not been reported, owing to the technical limitations of the statistical software. As such extreme values do not affect the decision regarding statistical significance, this conventional notation (*p* < 0.000001) was retained in the tables and text.

A priori sample-size calculation was performed using GPower 3.1.9.7 for repeated-measures analysis of variance with a three group × three time-point design. Based on published data from a similar biofeedback intervention for anxiety, where a very large effect was observed [19], we adopted a conservative estimate of a medium effect size f = 0.30. The following parameters were used: α = 0.05, power (1 β) = 0.80, number of groups = 3, number of measurements = 3, and sphericity correction ε = 1. The calculation yielded a required total sample size of N = 138. To account for an expected dropout rate of approximately 15%, we aimed to recruit at least 159 patients. The actual per-protocol sample exceeded this requirement, confirming adequate statistical power. The study was retrospectively registered at ClinicalTrials.gov (identifier: NCT07628153). Detailed protocols and statistical analysis plans are not publicly available. No interim analyses were planned or conducted, and no formal stopping rules were defined for this trial.

## 3. Results

**Figure 1:**
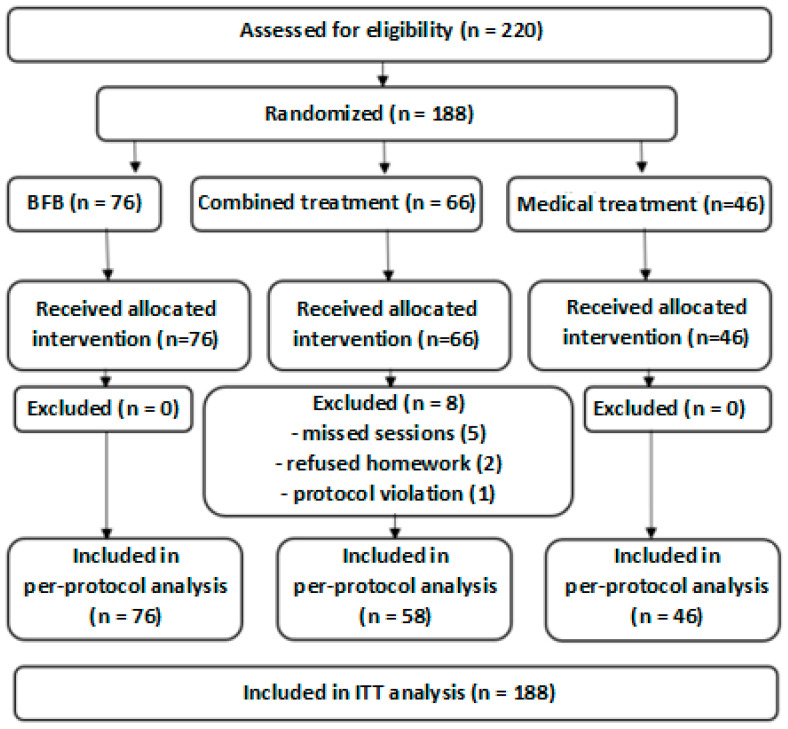
CONSORT flow diagram.

Figure 1 presents the CONSORT flow diagram detailing participant enrolment, allocation, follow-up, and analysis across the three study groups.

Adherence: In the BFB group, all 76 patients (100%) attended all 10 sessions. In the CT group, 58 completers attended a mean of 9.2 sessions (range 8–10). In the MT group, 44 of the 46 patients (95.7%) received ≥80% of the prescribed escitalopram dose (pill count). No other protocol deviation was observed.

### 3.1. Baseline Comparability and Statistical Analysis Strategy

In the first stage of the analysis, the comparability of the groups regarding the main relevant parameters was assessed: psychophysiological correlates of anxiety (heart rate and alpha rhythm magnitude in occipital leads), anxiety severity according to the HAM-A, and total mindfulness score according to the FFMQ. The Kruskal–Wallis H-test was used, and the level of statistical significance for almost all analyzed parameters exceeded 0.05, indicating no statistically significant differences between the groups at the start of the study. Table 1 presents the results.

To evaluate the comparative effectiveness of mindfulness formation using biofeedback to treat anxiety disorders, repeated-measures MANOVA was applied. As the dependent variables were measured on different scales (scores, percentages, and beats per minute), direct inclusion in the multivariate analysis could have led to the undesirable dominance of indicators with greater variability. To eliminate this effect and ensure the equal contribution of each variable to the analysis, all indicators were preliminarily standardized using Z-transformation (subtracting the mean and dividing by the standard deviation) for the combined sample. This transformation does not alter the correlation structure among variables but ensures that each indicator contributes equally to the multivariate solution, thereby preventing variables with larger variance from disproportionately influencing the results. This allowed us to bring the data to a single scale, while preserving the structure of interindividual differences and correlations between variables. This analysis assessed how a set of parameters (anxiety level, mindfulness characteristics, and psychophysiological indicators) changed over time (pre-treatment, 10 days post-treatment, and one-month follow-up) in the three groups with different treatment types.

### 3.2. Overall and Differentiated Parameter Dynamics

The overall pattern of change in all parameters during the study period was significantly different between groups (Wilks’ λ = 0.338; F(48, 308) = 4.617; *p* < 0.000001; multivariate partial η^2^ = 0.419). This indicates that the chosen therapy strategy had different effects not only on symptoms but also on associated psychological and physiological processes, and these differences manifested both immediately after treatment and in the long-term.

The subsequent use of univariate repeated-measures ANOVA for each dependent variable allowed us to identify the specific areas in which the effectiveness of the three therapeutic approaches differed. The pattern of change over time differed significantly between the groups for the vast majority of studied parameters. The only exception was heart rate (F(4, 352) = 0.949; *p* = 0.436), the dynamics of which were similar across all groups. The most pronounced differences in dynamics were recorded in the mindfulness domain. Its aspects, such as the ability to verbally describe inner experiences, engagement in the present moment, the ability not to react automatically to negative experiences, and the overall level of mindfulness, changed differently over time depending on the treatment received (F(4, 352) from 7.531 to 40.395; all *p* < 0.000001). Significant intergroup differences in dynamics were also identified for the overall level of anxiety and its components, somatic and psychic manifestations (F(4, 352) from 5.918 to 6.749; *p* = 0.0001 for somatic, *p* = 0.000038 for psychic, and *p* = 0.000030 for total anxiety). Among the psychophysiological parameters, a statistically significant effect of treatment type on changes over time was found for the alpha rhythm of the brain (F(4, 352) = 6.969; *p* = 0.000021), indicating a differential influence of therapy on the neurophysiological correlates of anxiety. The largest effect sizes were observed for mindfulness parameters (partial η^2^ ranging from 0.078 to 0.313), indicating a strong influence of treatment type on the dynamics of these variables. The results are presented in Table 2.

### 3.3. Detailed Effects Across Key Domains

To detail the significant interaction effects, post hoc analysis with Scheffé correction was performed. Analysis of the overall anxiety level dynamics showed significant within-group changes from baseline to post-treatment in all three groups. In the BFB group, the mean HAM-A score decreased from 26.55 (95% CI: 25.14–27.96) to 14.87 (95% CI: 13.60–16.13); *p* < 0.000001. In the CT group, it decreased from 26.48 (95% CI: 24.91–28.06) to 16.91 (95% CI: 15.39–18.44); *p* < 0.000001. In the MT group, it decreased from 26.85 (95% CI: 24.88–28.82) to 21.17 (95% CI: 18.83–23.51); *p* = 0.00013. At post-treatment, the MT group had significantly higher anxiety scores than the BFB group (mean difference = 6.30, 95% CI: 3.89 to 8.72, Cohen’s d = 0.94, 95% CI: 0.57 to 1.31, *p* = 0.00046), while being moderately but significantly different from the CT group (mean difference = 4.26, 95% CI: 0.60 to 6.92, Cohen’s d = 0.62, 95% CI: 0.24 to 1.00, *p* = 0.016). At one-month follow-up, the MT group had significantly higher anxiety scores than the CT group (20.50 [95% CI: 18.57–22.43] vs. 15.31 [95% CI: 13.55–17.07]; mean difference = 5.19, 95% CI: 2.60 to 7.78, Cohen’s d = 0.81, 95% CI: 0.42 to 1.20, *p* = 0.027), while the difference with the BFB group was *p* = 0.074 (20.50 [95% CI: 18.57–22.43] vs. 16.04 [95% CI: 14.70–17.38]; mean difference = 4.46, 95% CI: 2.20 to 6.72, Cohen’s d = 0.74, 95% CI: 0.37 to 1.10). The differences between the BFB and CT groups were not significant at any stage (mean difference = 0.73, 95% CI: −1.43 to 2.88, Cohen’s d = 0.12, 95% CI: −0.22 to 0.46, *p* > 0.89). Thus, both BFB and CT showed comparable and significant efficacy in reducing anxiety, whereas MT alone was less effective, although it demonstrated significant within-group reduction.

For mindfulness parameters (FFMQ total score), post hoc analysis with Scheffé correction showed that within-group changes from baseline to post-treatment were significant in the BFB and CT groups but not in the MT group. In the BFB group, the mean FFMQ score increased from 118.16 (95% CI: 114.58–121.74) to 141.24 (95% CI: 137.41–145.07); *p* < 0.000001. In the CT group, from 113.14 (95% CI: 109.55–116.73) to 128.93 (95% CI: 125.83–132.03); *p* < 0.000001. In the MT group, the mean score decreased from 119.09 (95% CI: 115.12–123.05) to 110.96 (95% CI: 107.92–113.99); *p* = 0.0465. Post-treatment, the mindfulness level in the MT group was significantly lower than that in the BFB group (110.96 vs. 141.24; mean difference = 30.28, 95% CI: 23.91 to 36.65, Cohen’s d = 2.20, 95% CI: 1.75 to 2.65, *p* < 0.000001) and the CT group (110.96 vs. 128.93; mean difference = 17.97, 95% CI: 11.25 to 24.70, Cohen’s d = 1.62, 95% CI: 1.17 to 2.07, *p* < 0.000001). The differences between the BFB and CT groups were also significant (141.24 vs. 128.93; mean difference = 12.31, 95% CI: 6.36 to 18.25, Cohen’s d = 0.83, 95% CI: 0.48 to 1.18, *p* = 0.00067). At the one-month follow-up, the MT group maintained significantly lower mindfulness scores compared to the BFB group (111.74 [95% CI: 108.99–114.49] vs. 136.29 [95% CI: 133.23–139.35]; mean difference = 24.55, 95% CI: 19.18 to 29.92, Cohen’s d = 2.12, 95% CI: 1.68 to 2.56, *p* < 0.000001) and compared to the CT group (111.74 vs. 131.17 [95% CI: 128.32–134.02]; mean difference = 19.43, 95% CI: 13.76 to 25.11, Cohen’s d = 1.94, 95% CI: 1.47 to 2.41, *p* < 0.000001), whereas differences between the BFB and CT groups were not significant (136.29 vs. 131.17; mean difference = 5.12, 95% CI: −1.10 to 10.13, Cohen’s d = 0.42, 95% CI: −0.03 to 0.87, *p* = 0.775). BFB and CT led to a significant increase in mindfulness, whereas monotherapy was associated with lower mindfulness.

Analysis of the mindfulness subscales revealed a similar but more differentiated pattern.

For the Observing subscale, within-group changes from baseline to post-treatment were significant in the BFB (*p* < 0.000001) and CT (*p* < 0.000001) groups, but not in the MT group (*p* = 0.999). Post-treatment, the Observing score in the MT group was significantly lower than that in the BFB (*p* = 0.00022) and CT (*p* = 0.0057) groups; the differences between BFB and CT were also significant (*p* < 0.000001). At one-month follow-up, the MT group’s score remained significantly lower than those of the BFB (*p* = 0.0051) and CT (*p* = 0.00038) groups, whereas the differences between the BFB and CT groups became non-significant (*p* = 0.997).

For the Describing subscale, a significant increase was observed in the BFB group (*p* = 0.0093), whereas changes in the CT group were not significant (*p* = 0.872) and the MT group showed a significant decrease (*p* < 0.000001). Post-treatment, the MT group was inferior to the BFB group (*p* < 0.000001) and the CT group (*p* = 0.0057); differences between BFB and CT groups were not significant (*p* = 0.9999). At the one-month follow-up, the patterns persisted: MT vs. BFB (*p* < 0.000001), MT vs. CT (*p* = 0.0126), and BFB vs. CT (*p* = 0.9999).

For the Acting with Awareness subscale, significant increases were observed in the BFB (*p* < 0.000001) and CT (*p* = 0.0029) groups, whereas a significant decrease was observed in the MT group (*p* < 0.000001). Post-treatment, the MT group was worse than the BFB (*p* < 0.000001) and CT (*p* < 0.000001) groups; the BFB and CT groups did not differ significantly (*p* = 0.9999). At the one-month follow-up, trends in differences remained: MT vs. BFB (*p* = 0.0011), MT vs. CT (*p* = 0.00004), and BFB vs. CT (*p* = 0.954).

For the Non-judgment subscale, significant increases were observed in the BFB (*p* < 0.000001) and CT (*p* < 0.000001) groups. However, the changes in the MT group were not statistically significant (*p* = 0.9995). Post-treatment, the MT group was inferior to the BFB group (*p* < 0.000001) but did not differ from the CT group (*p* = 0.9995); differences between the BFB and CT groups were not significant (*p* = 0.9998). At the one-month follow-up, differences between MT and BFB, and between MT and CT, became non-significant (*p* = 0.104 and *p* > 0.999, respectively); BFB and CT did not differ (*p* = 0.9999). Thus, the advantage of BFB over MT on this subscale is temporary.

For the Non-reactivity subscale, significant increases were observed in the BFB (*p* < 0.000001) and CT (*p* < 0.000001) groups; however, the changes in the MT group were not statistically significant (*p* = 0.9999). Post-treatment, the MT group was inferior to the BFB (*p* = 0.0015) and CT (*p* = 0.0276) groups; BFB and CT did not differ (*p* = 0.9999). At one-month follow-up, differences persisted: MT vs. BFB (*p* = 0.00019), MT vs. CT (*p* = 0.00007), and BFB vs. CT (*p* = 0.9999).

Thus, for all subscales except Non-judgment, the BFB and CT groups consistently outperformed MT both immediately after treatment and at the one-month follow-up. For Non-judgment, the effect of BFB was significant only after treatment.

The analysis of alpha rhythm dynamics showed that the course of BFB (alone or in combination with medication) induced a sharp increase in this parameter. In the BFB group, the mean alpha rhythm increased from 28.30 (95% CI: 24.77–31.83) to 47.28 (95% CI: 43.36–51.20); *p* < 0.000001. In the CT group, it increased from 29.52 (95% CI: 25.76–33.27) to 46.20 (95% CI: 42.12–50.28); *p* < 0.000001. In the MT group, an increase was also observed (from 27.56 [95% CI: 23.11–32.01] to 33.38 [95% CI: 28.52–38.24]), but the difference remained as not statistically significant (*p* = 0.408). Immediately after treatment, the alpha rhythm parameter in the MT group was significantly lower than that in the BFB (33.38 vs. 47.28; *p* = 0.0068) and CT (33.38 vs. 46.20; *p* = 0.0392) groups. The difference between the BFB and CT groups was not significant (47.28 vs. 46.20%, *p* = 0.9999).

One month after treatment, the groups that received BFB showed a substantial decrease in alpha rhythm compared to the post-intervention peak of 36.13 (95% CI: 31.93–40.32), and in the BFB group it was 39.74 (95% CI: 36.04–43.44). In the MT group, this parameter remained almost the same (33.22 [95% CI: 28.47–37.96]). Consequently, at one-month follow-up no statistically significant intergroup differences were found: MT vs. BFB (*p* = 0.998), MT vs. CT (*p* = 0.831), and BFB vs. CT (*p* = 0.989).

Thus, BFB training induced a strong but relatively short-lived increase in alpha rhythm, which subsided after the one-month follow-up. Monotherapy did not lead to significant changes in this parameter at any stage.

**Figure 2:**
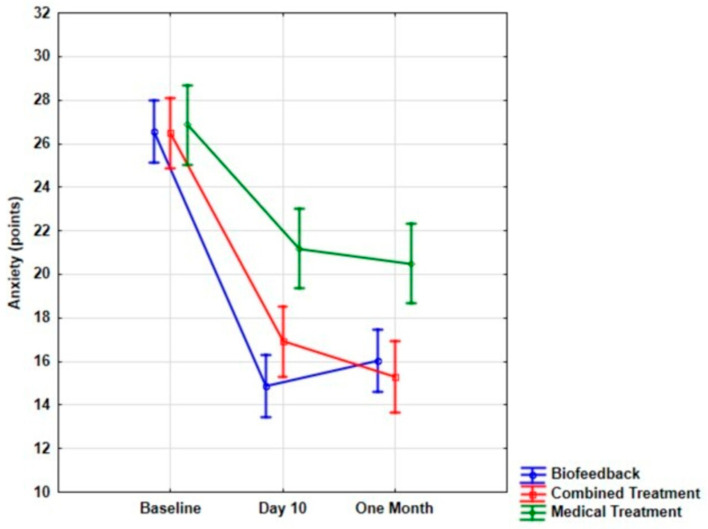
Average anxiety scores in groups with different treatment strategies for initial, control, and delayed measurement.

**Figure 3:**
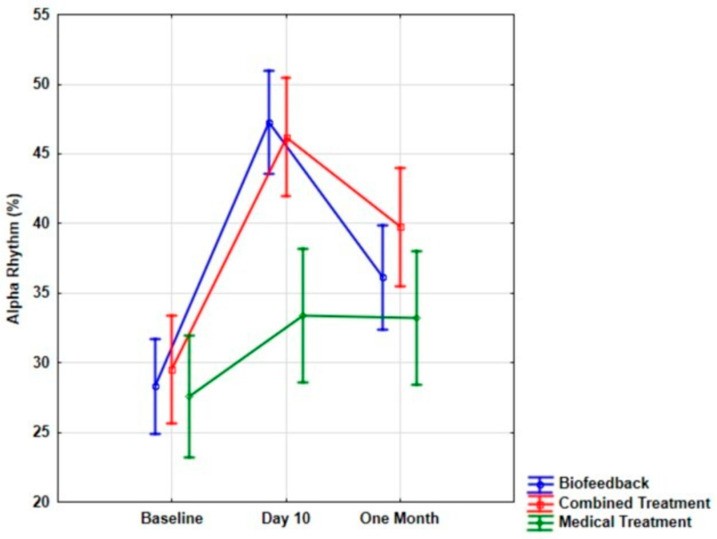
Average alpha rhythm in groups with different treatment strategies for initial, control, and delayed measurement.

**Figure 4:**
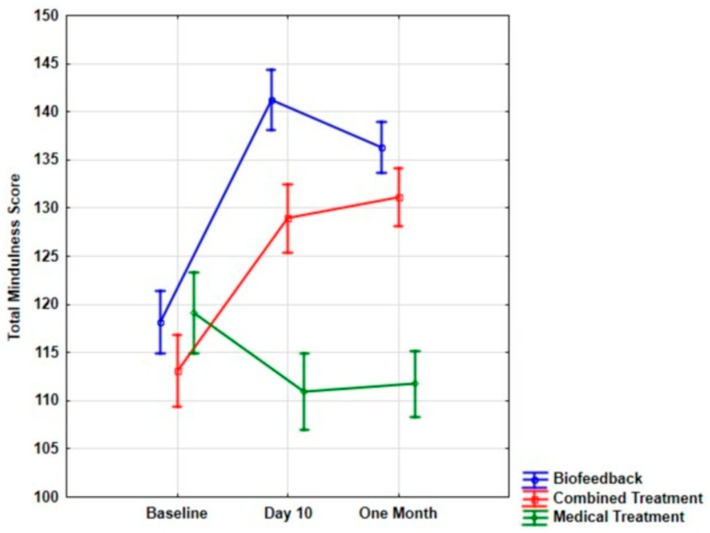
Average mindfulness scores in groups with different treatment strategies for initial, control, and delayed measurement.

The graphs below present the dynamics of the mean values of key indicators (anxiety, total mindfulness score, and alpha rhythm) in the original units of measurement across the three groups at three time points (Figure 2, Figure 3 and Figure 4).

Thus, the ANOVA results confirmed that different therapy strategies (BFB, combined, and medication) had a differential impact not only on the level of anxiety, but also on a set of associated psychophysiological correlates and cognitive-affective parameters, primarily mindfulness skills. The most favorable and comprehensive dynamics affecting both symptomatic and personal resource levels were observed in the BFB training group.

### 3.4. Sensitivity Analysis (Intention-to-Treat Principle)

To assess the impact of excluding eight participants from the CT group on the primary outcomes (reduction in anxiety measured by the HAM-A and increase in mindfulness measured by the FFMQ), we performed an ITT analysis on all 188 randomized patients. For the eight dropouts from the CT group (worst-case scenario), their post-treatment and one-month follow-up values were set to their baseline values; the remaining 180 patients contributed to the actual data.

Repeated-measures ANOVA for the total HAM-A score confirmed a significant group × time interaction (F(4, 370) = 6.65, *p* = 0.000035, η^2^ = 0.067), similar to that of the complete analysis (F(4, 352) = 6.749, *p* = 0.000030, η^2^ = 0.071). Within-group reductions from baseline to post-treatment were significant in all three groups: BFB from 26.55 to 14.87 points (*p* < 0.000001), CT from 25.83 to 17.42 (*p* < 0.000001), and MT from 26.85 to 21.17 (*p* = 0.00016). Post-treatment, the MT group was inferior to the BFB group (*p* = 0.00049) but did not differ from the CT group (*p* = 0.298); BFB and CT did not differ from each other. After one month, the differences between the MT and both active groups became non-significant (*p* = 0.077 and *p* = 0.093, respectively), which only weakened the MT vs. CT difference compared with the completer analysis (*p* = 0.027); however, the overall superiority of BFB and CT over MT remained.

The ITT-ANOVA for the FFMQ total score also showed a significant interaction (F(4, 370) = 44.78, *p* < 0.000001, η^2^ = 0.326), consistent with the complete analysis (F(4, 352) = 40.395, *p* < 0.000001, η^2^ = 0.313). All three groups showed significant changes from baseline to post-treatment: BFB from 118.16 to 141.24 points (*p* < 0.000001), CT from 109.18 to 123.06 (*p* < 0.000001), and MT from 119.09 to 110.96 (*p* = 0.045). Post-treatment, the MT group was worse than the BFB (*p* < 0.000001) and CT (*p* = 0.046) groups, and the BFB group was superior to the CT group (*p* < 0.000001). At the one-month follow-up, MT remained below the BFB (*p* < 0.000001) and CT (*p* = 0.015) groups; differences between BFB and CT also became significant (*p* = 0.023) under the worst-case imputation. However, as this comparison was non-significant in the completer analysis, this likely reflects an imputation artifact rather than a genuine strengthening.

Thus, ITT analysis with worst-case imputation confirmed the primary conclusion that BFB and CT are superior to medication monotherapy in reducing anxiety and developing mindfulness. The exclusion of eight CT patients did not distort the overall picture.

### 3.5. Adverse Events

During the inpatient stay, two patients in the MT group reported mild sedative effects that did not require treatment discontinuation. No other adverse events were observed in any of the three groups during the treatment period or at the one-month follow-up.

## 4. Discussion

The results demonstrated that a short-term mindfulness formation program using BFB, both as a monotherapy and in combination with pharmacotherapy, led to a significant reduction in anxiety symptoms which was maintained at one month, surpassing the effects of medication alone. These data are consistent with a large body of research confirming the effectiveness of BFB in anxiety and psychosomatic disorders [14,20,21]. The key mechanism explaining this effect lies in overcoming “physiological blindness”: BFB makes involuntary autonomic reactions (increased heart rate, decreased heart-rate variability, desynchronization of alpha rhythm) accessible to conscious perception and control [11,13]. By gaining direct sensory access to their internal states, patients empirically discover the connection between cognitive-emotional processes and bodily reactions, which is the foundation for developing self-regulation skills [12].

The most significant and comprehensive change was observed in the mindfulness domain. The BFB group showed a marked increase in the key components of the five-factor model, such as “Describing” (verbalization of experience) and “Acting with Awareness” (counteracting automaticity). This can be explained by the specifics of BFB training: the need to track changes on the screen and link them to internal efforts directly trains the skills of observation and mindful presence in the present moment. It is also necessary to take into account the possibility of the installation effect, with an open design potentially affecting the result. The techniques used in the program (breathing exercises, proprioceptive exercises, and audio meditations) are aimed at precisely developing these aspects of mindfulness, which correlates with data on the effectiveness of mindfulness-based therapies for developing observation and mindful actions [8,22].

An important neurophysiological correlate of the identified changes is the alpha rhythm dynamics. In the groups that included BFB training (BFB and CT), a significant increase in power was observed, whereas in the MT group, the indicator remained stable without significant changes. The alpha rhythm (8–13 Hz) is traditionally associated with a state of relaxed wakefulness, internal focus, and reduced anxiety [23]. Its increase during BFB may reflect a general decrease in cortical hyperexcitability, a characteristic of anxiety, and the development of focused internal attention skills, a key element of mindfulness [24]. In addition, repeated successful acts of self-regulation, reinforced by feedback, contribute to the formation of new, more adaptive patterns of neuronal activity [13,25]. At the same time, the increase in alpha rhythm power was short-lived and decreased after 1 month of follow-up, which indicates a direct physiological effect of BFB training, but longer-term studies are needed to assess whether repeated training can cause stable neurophysiological adaptation.

Comparison with the CT group showed comparable efficacy in reducing anxiety; however, the BFB group demonstrated more pronounced positive dynamics in specific mindfulness components (“Describing”, “Acting with Awareness”). This may indicate that the non-pharmacological BFB approach targets the psychological mechanisms of skill development more directly, whereas pharmacotherapy primarily affects the neurochemical substrates of the symptoms. It should be noted, that the BFB program included multiple therapeutic elements. Therefore, the observed benefits cannot be attributed solely to biofeedback; they likely reflect a synergistic effect of the entire treatment package. The absence of negative mindfulness dynamics in the BFB group, unlike in the MT group, where there was no improvement, highlights the resource-based and developmental nature of the BFB intervention, which is crucial for the long-term sustainability of outcomes.

The BFB and CT groups had more therapist contact than the MT group, potentially influencing results via non-specific factors (e.g., therapeutic alliance). This should be considered when comparing interventions. While our results suggest that BFB-based training may reduce anxiety more rapidly than escitalopram in this inpatient sample, this should not be interpreted as evidence of overall superiority. The short treatment window, unequal therapist contact, and delayed SSRI onset all likely contributed to the observed difference. Thus, we view BFB as a promising adjunctive or alternative option, but head-to-head comparisons with longer follow-up are necessary.

### 4.1. Limitations

This study had several limitations. The open-label design could have introduced bias because patients and therapists knew the group allocation, although an independent, blinded psychiatrist performed the anxiety assessment (HAM-A). In this regard, the results based on blind analysis should be considered more reasonable. The results have limited generalizability to outpatients and other subtypes of anxiety disorders as the study included only inpatients with a first-time diagnosis treated at a single center. Although the Scheffé test was used for post hoc pairwise comparisons, no global correction was applied to multiple secondary outcomes. Therefore, some significant effects among the secondary outcomes may be due to chance, and these results should be considered preliminary. The trial was registered retrospectively and a detailed protocol was not publicly available, which may introduce reporting bias. Nevertheless, these findings support the potential of BFB training and provide a basis for future studies.

All eight dropouts occurred in the combined therapy group. However, the baseline characteristics of dropouts did not differ from completers, and the ITT analysis using worst-case imputation yielded similar conclusions, suggesting that attrition bias is unlikely to have altered our primary findings. Nevertheless, this pattern should be considered when interpreting the results.

We acknowledge that the BOCF imputation is conservative; future studies could employ multiple imputation or mixed models.

We did not record specific subtypes of anxiety disorders (e.g., GAD, panic disorder, phobic disorders). Given that treatment response may vary across subtypes, this limits the generalizability of our findings to particular diagnostic groups.

Adverse events were assessed via spontaneous reports rather than systematic questionnaires, which may have under-reported mild or subjective side effects. Systematic monitoring should be implemented in future trials.

### 4.2. Future Directions

Further research should extend the follow-up period to 6 and 12 months to assess the stability of mindfulness skills and relapse rates. Future studies should also focus on differentiating the effectiveness of specific BFB protocols (EEG, cardio) for correcting deficient mindfulness components in different clinical-psychological subtypes of patients, as well as identifying predictors of therapy success to optimize treatment selection.

## 5. Conclusions

Consequently, the analysis conducted allowed us to draw the following conclusions:

1. A short-term (10-day) mindfulness formation program using biofeedback (BFB) showed promising results in reducing anxiety symptoms in this inpatient sample. Its effectiveness was comparable to that of CT and appeared superior to that of medication alone in this study. However, these findings should be interpreted with caution owing to the open-label design and other limitations.
2. BFB training has a comprehensive positive impact on the structure of mindfulness, contributing to the significant development of its key components, such as the ability to verbally describe inner experiences and mindful nonautomatic engagement in current activities.
3. Positive changes at the psychological level are accompanied by favorable neurophysiological dynamics, particularly a tendency to increase alpha rhythm power, which may reflect a reduction in cortical hyperexcitability and the development of internal focus skills.
4. The data obtained confirm that the psychological mechanism of BFB action in anxiety disorders is realized not only through the correction of autonomic imbalance, but also through the targeted development of mindfulness skills.

## Figures and Tables

**Table 1:** Comparability of groups by main relevant parameters.

Parameter	BFB (n = 76)	Combined Treatment (n = 58)	Medical Treatment (n = 46)	Significance of Differences
Heart rate (HR)	87.97 ± 12.3	84.99 ± 16.0	84.56 ± 15.0	0.23
Alpha rhythm magnitude	28.30 ± 15.5	29.52 ± 14.3	27.56 ± 15.0	0.67
Total HAM-A score	26.55 ± 6.2	26.48 ± 6.0	26.85 ± 6.6	0.86
Total FFMQ score	118.16 ± 15.7	113.14 ± 13.7	119.09 ± 13.3	0.08

**Table 2:** Effect of treatment type on changes in psychological and physiological parameters during the study and at follow-up (results of stepwise analysis).

Variable	F(4, 352)	*p*	Partial η^2^
Heart rate (HR)	0.949	0.436	0.011
Alpha rhythm	6.969	0.000021	0.073
Somatic anxiety (HAM-A)	5.918	0.0001	0.063
Psychic anxiety (HAM-A)	6.622	0.000038	0.070
Total anxiety (HAM-A)	6.749	0.000030	0.071
Observing	16.402	<0.000001	0.156
Describing	16.633	<0.000001	0.158
Acting with Awareness	20.489	<0.000001	0.188
Non-judgment	7.531	0.000008	0.078
Non-reactivity	16.345	<0.000001	0.156
Total Score of Mindfulness	40.395	<0.000001	0.313

## Data Availability

The data supporting the findings of this study are available from the corresponding author upon reasonable request, subject to institutional policies.

## Abbreviations

BFB	Biofeedback
CT	Combined therapy
FFMQ	Five Facet Mindfulness Questionnaire
HAM-A	Hamilton Anxiety Rating Scale
ITT	Intention-to-treat
MT	Medication therapy
